# Heterologous Expression of *MfWRKY7* of Resurrection Plant *Myrothamnus flabellifolia* Enhances Salt and Drought Tolerance in *Arabidopsis*

**DOI:** 10.3390/ijms23147890

**Published:** 2022-07-17

**Authors:** Zhuo Huang, Ling Liu, Linli Jian, Wenxin Xu, Jiatong Wang, Yaxuan Li, Cai-Zhong Jiang

**Affiliations:** 1 College of Landscape Architecture, Sichuan Agricultural University, Chengdu 611130, China; liuling@stu.sicau.edu.cn (L.L.); jianlinli@stu.sicau.edu.cn (L.J.); xuwenxin@stu.sicau.edu.cn (W.X.); wangjiatong@stu.sicau.edu.cn (J.W.); liyaxuanxx@stu.sicau.edu.cn (Y.L.); 2 Department of Plant Sciences, University of California Davis, Davis, CA 95616, USA; caizhong.jiang@usda.gov; 3 Crops Pathology and Genetics Research Unit, United States Department of Agriculture, Agricultural Research Service, Davis, CA 95616, USA

**Keywords:** *Myrothamnus flabellifolia*, WRKY, transcription factor, drought stress

## Abstract

Drought and salinity have become major environmental problems that affect the production of agriculture, forestry and horticulture. The identification of stress-tolerant genes from plants adaptive to harsh environments might be a feasible strategy for plant genetic improvement to address the challenges brought by global climate changes. In this study, a dehydration-upregulated gene *MfWRKY7* of resurrection Plant *Myrothamnus* *flabellifolia*, encoding a group IId WRKY transcription factor, was cloned and characterized. The overexpression of *MfWRKY7* in *Arabidopsis* increased root length and tolerance to drought and NaCl at both seedling and adult stages. Further investigation indicated that *MfWRKY7* transgenic plants had higher contents of chlorophyll, proline, soluble protein, and soluble sugar but a lower water loss rate and malondialdehyde content compared with wild-type plants under both drought and salinity stresses. Moreover, the higher activities of antioxidant enzymes and lower accumulation of O_2_^−^ and H_2_O_2_ in *MfWRKY7* transgenic plants were also found, indicating enhanced antioxidation capacity by MfWRKY7. These findings showed that MfWRKY7 may function in positive regulation of responses to drought and salinity stresses, and therefore, it has potential application value in genetic improvement of plant tolerance to abiotic stress.

## 1. Introduction

Abiotic stresses, such as drought, salinity and low temperature, are the main environmental factors negatively affecting plant growth and development [1]. Under long-term natural selection, plants have evolved extremely complex mechanisms to adapt to adverse environments through generating morphological, physiological and molecular responses. Transcription factors (TFs) are among the most important regulators in these processes. Many TFs families, such as WRKY, AP2/ERF and NAC, have been reported to play important and unique roles in responses to abiotic stress [2].

WRKY TFs belong to a plant-specific transcription factor family [3]. All WRKY proteins are featured by the highly conserved WRKY domain comprising of amino acids WRKYGQK at its N-terminus and the zinc-finger-like motifs of C-X4-5C-X22-23-H-X-H or C-X7-CX23-H-X-C at its C-terminus, which enable them to bind with W-box in the promoter region of target genes with a DNA sequence of (C/T)TGAC(C/T) [4]. According to the structure of the WRKY protein, they could be divided into three groups: members with two WRKY domains belong to group I, while members of groups II and III have only one WRKY domain [5,6].

In recent years, increasing evidence indicates that WRKYs are involved in various abiotic stress responses in plants, especially in responses to salt and drought stresses [7,8]. For example, the overexpression of *Eriobotrya japonica EjWRKY17*, wheat (*Triticum aestivum*) *TaWRKY75-A*, and bamboo (*Phyllostachys edulis*) *PheWRKY86* enhanced drought tolerance in transgenic *Arabidopsis* [9,10,11], and significantly enhanced salt tolerance was found in transgenic *Arabidopsis* overexpressing *Polygonum cuspidatum PcWRKY11*, sweetpotato (*Ipomoea batatas*) *IbWRKY2* and Tartary buckwheat (*Fagopyrum tataricum*) *FtWRKY46* [12,13,14]. Therefore, WRKY TFs are considered as the reservoir of regulators for abiotic stress responses and need to be further investigated.

WRKY transcription factor WRKY7 belongs to the subgroup IId of the WRKY family, and its main role is to regulate plant response to pathogens [15,16]. The heterologous expression of *BrWRKY7* from *Brassica rapa* enhanced resistance against bacterial soft rot caused by *Pectobacterium carotovorum* in *Arabidopsis* [17], while pathogen-induced WRKY7 transcription factor played a negative role in defense responses to *P. syringae* [18]. However, the involvement of WRKY7 in abiotic stress responses was seldom reported. Recently, Yang et al. [19] found that both *JrWRKY2* and *JrWRKY7* of *Juglans regia* mediate responses to abiotic stresses and abscisic acid (ABA) through the formation of homodimers and interaction. The overexpression of *JrWRKY2* and *JrWRKY7* improved plant tolerance to NaCl, PEG, abscisic acid (ABA) and cold stress. However, it remains unclear whether WRKY7 is involved in response to other plant abiotic stresses, such as drought [19].

The resurrection plant *Myrothamnus flabellifolia*, which is believed to be one of the most ancient plants on the planet, is widely used as African traditional medicine [20,21]. They are distributed throughout southern Africa in disjunct populations from Namibia to Tanzania, with the highest density of plants occurring in South Africa and Zimbabwe. It was known in isiZulu as Uvukakwabafile, which means ‘awake from the dead’. This might originate from its adaptability to extreme drought surroundings, which benefits from evolved powerful survival strategies including a well-developed root system and the capability to recover from dehydration [22,23,24]. However, few genes related to the drought tolerance of *M. flabellifolia* have been characterized, and the underlying molecular mechanisms are largely unknown. A previous study showed that many transcription factors were involved in the dehydration responses of *M. flabellifolia* by transcriptome sequencing, in which *MfWRKY7* is immediately upregulated in the early stage of dehydration, but its roles in stress responses are unclear [25]. In this study, we cloned *MfWRKY7* and overexpressed it in *Arabidopsis*. The drought and salt treatments were performed to evaluate its function in stress tolerance. Phenotype and physiological index analysis before and after treatments were compared, and the potential regulatory mechanisms were speculated and discussed.

## 2. Results

### 2.1. Isolation and Characterization of MfWRKY7

**Figure 1:**
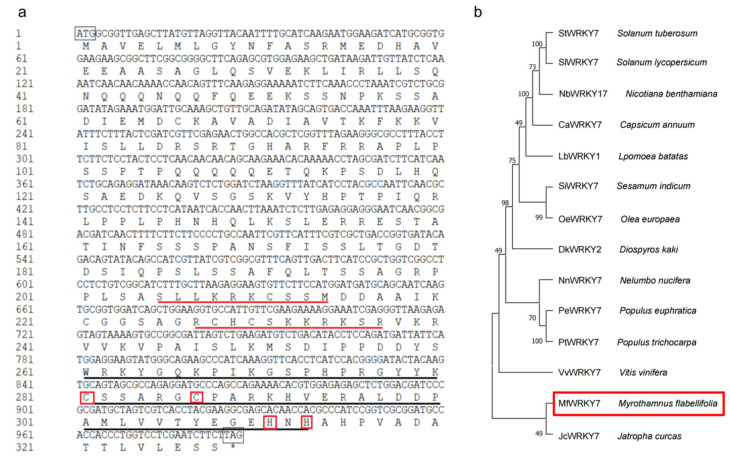
Sequence analysis of MfWRKY7. (**a**) The nucleotide and deduced amino acid sequences of MfWRKY7. Black boxes represented start codons and stop codons (also indicated by *), black line represented WRKY domain, red boxes represented zinc finger structure, and red line represented nuclear location signal, respectively. (**b**) Phylogenetic tree constructed using neighbor-joining method. MfWRKY7 was indicated by a red box. The GenBank accession numbers for the sequences used are listed in Supplementary Table S1.

The cDNA sequence of *MfWRKY7* was cloned from *M. flabellifolia* by PCR amplification. The length of nucleotide sequence of *MfWRKY7* is 987 bp, encoding 328 amino acid residues. The deduced protein has a predicted molecular weight of 36 kD and an isoelectric point (pI) of 9.88. Two nuclear localization signals (NLS) “SLLKRKCSSM” at 205aa and “RCHCSKKRKSR” at 227aa were predicted according to NLS-MAPPER program. SMART analysis indicated that MfWRKY7 contains a typical WRKY motif “WRKYGQK” locating at 261–267aa and a Cx5Cx23HxH-type zinc finger structure. (Figure 1a).

Multiple sequence alignment between MfWRKY7 and thirteen mostly homologous WRKY proteins indicated that MfWRKY7 belongs to the second class of the WRKY transcription factor family (Figure S1). The phylogenetic analysis revealed that the MfWRKY7 is most homologous to *Jatropha curcas* JcWRKY7, which was grouped to a separated clade (Figure 1b).

### 2.2. Overexpressing MfWRKY7 in Arabidopsis Increased Drought and Salt Tolerance

In order to investigate whether *MfWRKY7* is associated with drought and salt stress tolerance, heterologous expression of the *MfWRKY7* gene in *Arabidopsis* was performed by constructing a binary vector 35S::pGSA1403-MfWRKY7. T_1_ transgenic lines were acquired by kanamycin resistance screening, and two T_3_ homozygous transgenic lines obtained by PCR, OE1 and OE2, were randomly selected for further evaluation of abiotic stress tolerance. Quantitative real-time PCR (qRT-PCR) analysis indicated that *MfWRKY7* was expressed in OE1 and OE2, in which that of OE1 was slightly higher than that of OE2 (Figure S2).

**Figure 2:**
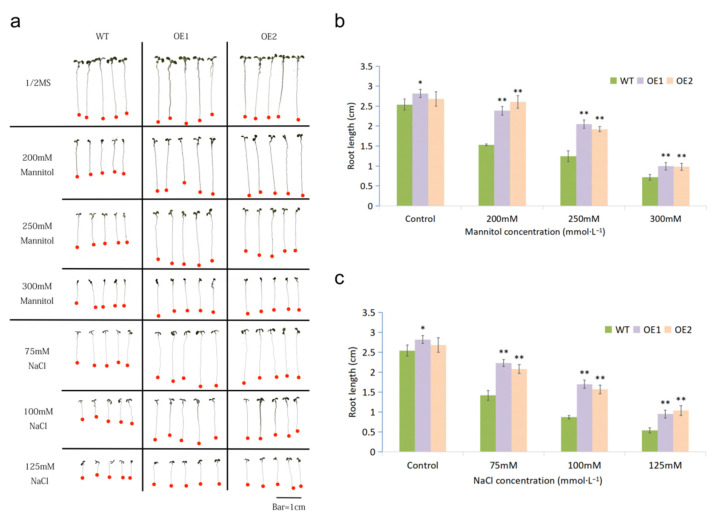
Analysis of drought and salinity tolerance at the seedling stage. (**a**) Morphology of transgenic and WT seedlings grown on 1/2 MS solid medium containing different contents of mannitol and NaCI. (**b,c**) The primary root length of WT and OE lines under conditions with or without mannitol and NaCl, respectively. Data were presented as mean and SD values of three independent experiments. The asterisk indicates a significant difference (* *p* < 0.05; ** *p* < 0.01, by Student’s *t*-test) comparing to WT.

We performed drought and salt treatments for plants at seedling and adult stages. At the seedling stage, treatments of artificially simulated drought stress by applying different concentrations of mannitol and salt stress by applying different concentrations of NaCl in the 1/2 solid MS medium were conducted. No significant differences of lateral roots number were observed between WT and transgenic plants under normal conditions. However, the root length of two OE lines, especially OE1, was longer than that of the WT. After treatments with mannitol and NaCl, root growth was inhibited in all plants. However, the OE1 and OE2 exhibited significantly longer roots than those of WT under all stress treatments (Figure 2a–c). Concurrently, a clearly larger leaf area was also observed in transgenic lines (Figure 2a).

**Figure 3:**
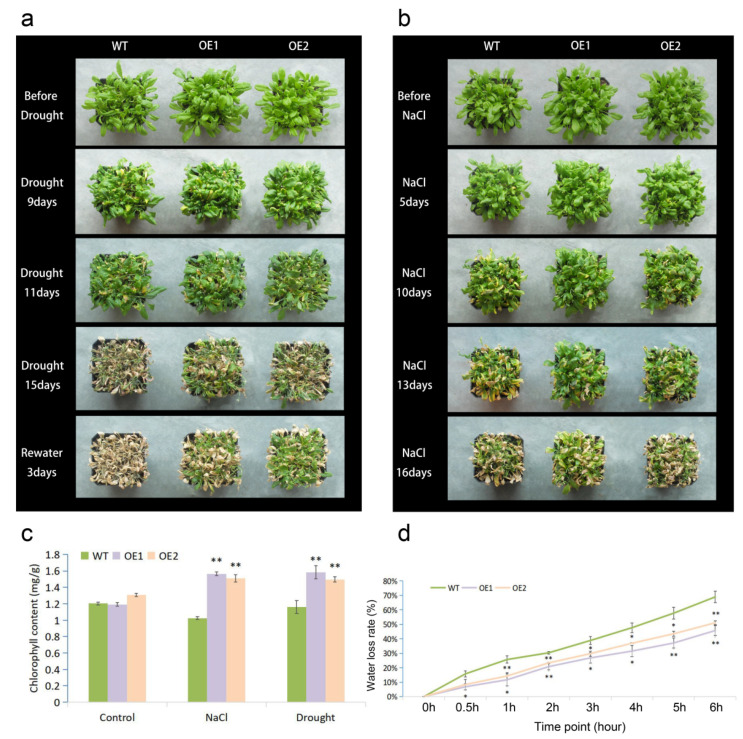
Analysis of drought and salinity tolerance at the adult stage. (**a**,**b**) showed the growth status of transgenic and WT plants during drought and salinity treatments, (**c**,**d**) showed measurements of chlorophyll content and water loss rate, respectively. The error bars are SDs, and the asterisk indicates a significant difference (* *p* < 0.05; ** *p* < 0.01).

At the adult stage, after being grown for 4 weeks in soil, WT and transgenic plants were treated with natural drought (withholding watering) and salt (irrigating with 300 mM NaCl solution) stresses. There was no obvious morphological difference between the transgenic and WT plants before treatments (Figure 3a,b). At 9 days of natural drought, the leaves of all lines were slightly withered; 15 days after withholding watering, most of the leaves in WT were withered and chlorotic, while OE1 and OE2 still maintained more green leaves; 3 days after rehydration, almost all WT plants died, while some transgenic plants survived (Figure 3a). After 10 days of NaCl treatment, the wilt symptoms were found on both WT and OE lines; after 13 days of salt treatment, the leaves of WT were seriously wilted, whereas most leaves of OE1 and OE2 were still green. At 16 days of salt treatment, more wilted leaves were detected, but the OE lines, especially line OE1, seemed to have a larger portion of green leaves (Figure 3b). In conclusion, transgenic plants overexpressing *MfWRKY7* showed better growth under both drought and salt stresses.

We measured the chlorophyll content in WT and OE lines. Under normal conditions, there was no significant difference in the chlorophyll content between WT and transgenic plants. After treatments of 300 mM NaCl solution and natural drought, the chlorophyll content in transgenic plants was increased and significantly higher than that in WT (Figure 3c). These results were consistent with that more green leaves were found in OE lines under drought and salt stresses. Water stress tolerance is closely related to the water loss rate (WLR). Therefore, we measured WLR under natural drought stress and found that both lines OE1 and OE2 exhibited significantly lower WLR during the treatment (Figure 3d).

### 2.3. MfWRKY7 Improved Osmotic Adjustment Ability in Transgenic Arabidopsis

**Figure 4:**
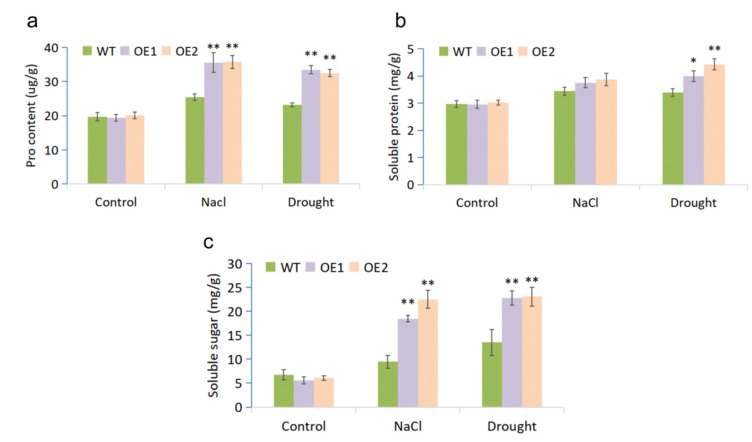
Evaluation of physiological indices responsive to stress. Proline content (**a**), soluble protein content (**b**) and soluble sugar content (**c**) were measured in OE lines and WT plants. Samples used for measurements were leaves of plants treated by drought (stopping watering 4-week-old plants for 10 days) and salt (irrigating 4-week-old plants using 300 mM NaCl solution for 7 days) stresses. Each measurement was performed in three replicates. The data are shown as mean and SD (bars) of three biological replicates, and the asterisk indicated the significant difference (* *p* < 0.05; ** *p* < 0.01).

Proline is involved in the regulation of osmotic potential in plants by participating in the maintenance of cellular water balance as well as avoiding ion toxicity. Both drought and salt stresses induced accumulations of proline in WT and OE lines, while the latter accumulated more (Figure 4a). Soluble sugar and soluble protein are also important osmotic adjustment substances. Comparing to control conditions, the soluble protein content of WT and OE lines was increased under both stresses. Under salt stress, the mean soluble protein contents of OE1 and OE2 were higher than that of WT; however, the difference was not statistically significant. Under drought treatment, both OE lines exhibited significantly higher soluble protein contents than did the WT (Figure 4b). Contents of soluble sugar in OE1 and OE2 were strongly upregulated by salt and drought stresses, and they were significantly higher than that of WT under both treatments (Figure 4c). These results indicated that the overexpression of *MfWRKY7* improved the osmotic adjustment ability of transgenic *Arabidopsis* under salt and drought stresses.

### 2.4. Effect of MfWRKY7 on Antioxidant Metabolism in Arabidopsis under Drought and Salinity Stresses

**Figure 5:**
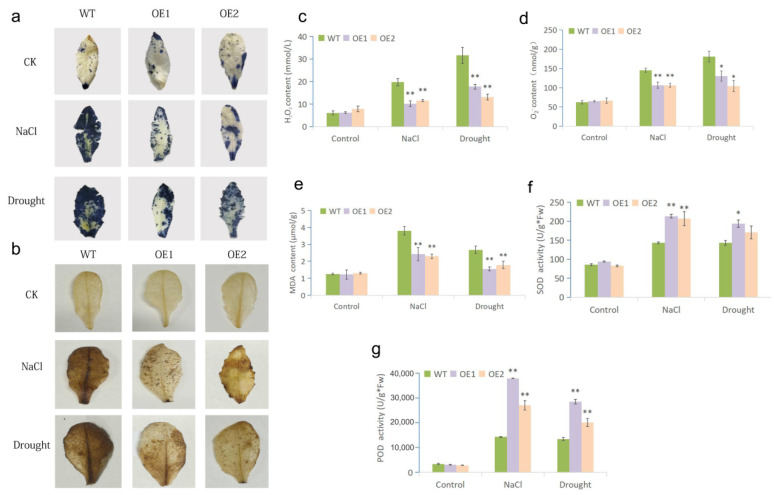
Analysis of oxidative damage and ROS scavenging ability under drought and salt treatments. Histochemical staining with DAB (**a**) and NBT (**b**) were used to detect the accumulation of H_2_O_2_ and O_2_^−^. (**c**,**d**) showed the quantification of H_2_O_2_ and O_2_^−^ contents of OE and WT plants before and after stress treatments, respectively. (**e**–**g**) indicated the MDA content and activities of SOD and POD in the leaves of OE and WT plants, respectively. Samples used for staining and measurements were leaves of plants treated by drought (stopping watering 4-week-old plants for 10 days) and salt (irrigating 4-week-old plants using 300 mM NaCl solution for 7 days) stresses. Each measurement was performed in three replicates. Data were presented as mean and SD values of three independent experiments. Asterisks indicated significant difference (* *p*  <  0.05, ** *p*  <  0.01, by Student’s *t*-test) between WT and OE lines.

When plants are subjected to abiotic stress, an excessive accumulation of reactive oxygen species (ROS), such as hydrogen peroxide (H_2_O_2_) and superoxide anion radicals (O_2_^−^), can cause oxidative damage to biomolecules. We used histochemical staining by 3,3′-diaminobenzi dine (DAB) and nitroblue tetrazolium (NBT) to detect cellular reactive oxygen species levels under drought and salinity stresses. As shown in Figure 5a,b, before stress treatment, leaves of both WT and transgenic plants were only stained in a very small part by DAB (deep blue), and they were almost not stained by NBT. Under both drought and salinity stresses, the larger leaf area of WT could be stained deeper comparing to those of OEs (Figure 5a,b). Consistently, significantly lower H_2_O_2_ and O_2_^−^ levels were detected in transgenic lines under both stresses (Figure 5c,d). These results indicated that less ROS was accumulated in the OE lines under drought and salt stresses.

Malondialdehyde (MDA) accumulation represents the degree of cell membrane lipid peroxidation and the intensity of plant response to stressful circumstances. Although drought and salt treatments both increased the MDA content in all plants, OE1 and OE2 remained at considerably lower levels than in WT (Figure 5e). The less MDA accumulation in OE lines indicated that the degrees of membrane peroxidation and plasma membrane damage were lower in OE plants.

The antioxidant enzymes, such as superoxide dismutase (SOD) and peroxidase (POD), are key enzymes to scavenge ROS. Compared with WT plants, the activities of SOD and POD in OE lines were significantly increased under drought and salt treatments (Figure 5f,g), which was consistent with the lower level of ROS.

### 2.5. MfWRKY7 Promoted Stomatal Closure Induced by Drought

**Figure 6:**
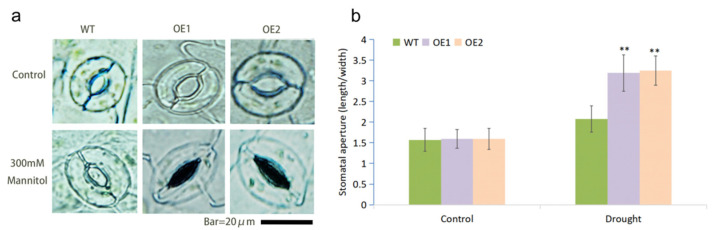
Measurements of stomatal aperture. (**a**) Microscopy observation of stomatal aperture of *MfWRKY7* transgenic and WT plants in response to 300 mM mannitol. (**b**) Measurement of stomatal aperture with or without mannitol treatment. Data were presented as mean and SD values of three independent experiments. Asterisks indicated significant difference (** *p*  <  0.01, by Student’s *t*-test) between WT and OE lines.

Stomatal movement plays a central role in transpiration adjustment upon drought stress. We analyzed stomatal closure in leaves of WT and transgenic plants under 300 mM mannitol treatment. The stomata of both WT and OE lines were fully open under normal circumstance with a stomatal aperture of about 1.5. After mannitol treatment, the stomatal closure was found in both OE and WT plants (Figure 6a). The stomatal apertures of OE1 and OE2 were increased to about 3.2, which was significantly higher than the 2.0 of WT (Figure 6b). This result showed that the overexpression of *MfWRKY7* facilitated stomatal closure under drought.

## 3. Discussion

Since the isolation of the first WRKY TF from sweet potato [26], it has been widely reported that WRKY transcription factors in plants play a critical role in abiotic stress responses [27,28]. In this study, we isolated a dehydration-inducible WRKY gene *MfWRKY7* from *M*. *flabellifolia*. Sequence analysis illustrated that MfWRKY7 contains a highly conserved WRKY motif of ‘WRKYGQK’ and a C2H2 zinc finger structure (Figure 1a). Phylogenetic analysis showed that MfWRKY7 is highly homologous to JcWRKY7 of *Jatropha curcas*, which is native to tropical America and is a stress-tolerant plant being advocated for growing on wastelands [29]. The overexpression of *JcWRKY* in tobacco enhanced salinity resistance in transgenic plants [30]. Similarly, our results showed that the overexpression of *MfWRKY7* can significantly increase the tolerance of transgenic *Arabidopsis* to drought and salt stresses. These results suggest that plant species adapting to wastelands and extremely drought environments, such as *M*. *flabellifolia*, can be exploited and utilized as an important stress-tolerant genetic resources.

Under adverse environmental conditions, enhancing the growth of primary roots would offer an advantage to the plants by expanding their domains of water supply [31]. Therefore, root length is an important indicator of drought tolerance. *CsWRKY7* of *Camellia sinensis* responded to NaCl, mannitol, PEG, and diverse hormones treatments. Seedlings of transgenic *Arabidopsis* overexpressing *CsWRKY7* showed longer primary roots than the WT. However, no significant difference in root growth was observed between WT and the transgenic lines in the presence of different stresses [32]. In our study, we found that the root length of OE lines was longer than that of WT under normal conditions. Such a difference is more significant under drought and salt treatments (Figure 2). These results indicated that MfWRKY7 promotes root growth under both normal condition and stress treatments. Furthermore, the OE lines displayed higher chlorophyll content than WT at the adult stage. The chlorophyll content is positively correlated to photosynthetic rate, and also highly associated with improved transpiration efficiency under water-limited conditions [33,34,35]. These results indicated that MfWRKY7 may play a positive regulatory role in maintaining the stability of chlorophyll content to ensure plants have relatively normal growth under stress.

Transpirational water loss through the stomata is a key determinant of drought tolerance [36]. The rational regulation of stomatal movement and aperture size will help to keep water balance in vivo under water-deficit conditions [37]. Here, we found that the stomata apertures of *MfWRKY7* OE plants were significantly higher than that of WT when treated by 300 mM mannitol. This result indicated that MfWRKY7 may increase the sensitivity of stomatal movement to water conditions and promote its closure under a water deficit. This is also supported by the result that the OE lines exhibited a significantly lower water loss rate compared to WT. Thus, the promoted root growth and stomata movement as well as the decreased WLR brought by the introduction of MfWRKY7 may lead to better water uptake and retention abilities under water-deficit conditions and hence higher tolerance.

Plants can produce osmoregulatory substances, including proline, soluble proteins and soluble sugars, under stress. Proline can serve as an ROS scavenger [38,39], while soluble proteins and soluble sugars mitigate dehydration stress and help maintain the macromolecules structure and function [40]. In our data, the levels of these three osmoregulatory substances were significantly increased after drought and salinity stresses (Figure 4). The results showed that *MfWRKY7* overexpression enhanced the osmoregulation ability of transgenic lines under stress. *CsWRKY7* gene expression was induced by various osmotic stresses. However, the overexpression of *CsWRKY7* in *Arabidopsis* did not enhance tolerance to osmotic stress [32]. JrWRKY7 was induced by NaCl and polyethylene glycol (PEG). The overexpression of *JrWRKY7* improved plant tolerance to NaCl, PEG, abscisic acid, and cold stress [19]. These results indicated that different WRKY7 TFs may have divergent functions.

A stress-induced excessive accumulation of ROS causes oxidative damage in plants [41]. Therefore, the ability of scavenging ROS is essential for stress tolerance. By *MfWRKY7* overexpression, the oxidative stress of OE lines is significantly lower than those of WT, which was indicated by a lower degree of lipid peroxidation (lower MDA accumulation) (Figure 5e) and ROS accumulation (O_2_^−^ and H_2_O_2_ contents) (Figure 5a–d) under both drought and salt treatments. It is well known that antioxidant enzymes, such as SOD, POD and CAT, have important roles in scavenging ROS and promoting plant tolerance to abiotic stresses [42,43]. In our study, there was no obvious difference of activities of SOD and POD before treatment between WT and OE plants, whereas those in OE plants were significantly higher than WT plants after drought and NaCl treatments (Figure 5e,f). The data demonstrated that *MfWRKY7* overexpression effectively activated the antioxidant system in transgenic *Arabidopsis* lines to reduce the ROS-induced oxidative damage and maintain the dynamic balance of ROS, thereby improving tolerance to drought and salinity stresses. Previous studies showed that overexpressing *CmWRKY10, SbWRKY30,* or *MfWRKY17* could enhance the activities of antioxidant enzymes and hence stress tolerance [44,45,46]. Therefore, strengthening the antioxidant system might be a generally common strategy of WRKY TFs to increase plant’s tolerance to abiotic stress. It is worth further study on which pathways *MfWRKY7* works through to enhance root elongation and the antioxidation system. MfWRKY7 might be potentially valuable for the genetic improvement of drought and salt tolerance in economically important plants, such as crops and ornamental plants.

## 4. Materials and Methods

### 4.1. Plant Materials and Growth Conditions

The wild-type *Arabidopsis* (Col-0, WT) and *M. flabellifolia* used in this study were provided by the Department of Plant Sciences of the University of California, Davis. *M. flabellifolia* plants were grown under conditions of 12 h light/12 h dark at temperatures of 22 °C/18 °C, respectively, with a relative air humidity of 60%.

Seeds of WT and OE lines were sterilized with diluted bleach solution for 5 min and washed 3–5 times with sterilized deionized water. Seeds were placed on plates containing 1/2 Murashige and Skoog (MS) solid medium with pH ≈ 6.0. After 2 days of stratification at 4 °C, plates were transferred in an illuminated incubator for about 10 days. Seedlings were transplanted into pots with soil and vermiculite (1:1) as the cultivation substrate and were grown for 4 weeks under condition of 16 h light/8 h dark, 24 °C/22 °C, and 75% relative humidity.

### 4.2. Cloning and Sequence Analysis of MfWRKY7

Total RNA was extracted from fresh leaves of *M. flabellifolia* using the Plant Total RNA Isolation Kit (LANBO, Chengdu, China). The first strand of cDNA was then synthesized with reverse transcriptase Reverse Transcriptase M-MLV (RNaseH-) (TaKaRa, Dalian, China) under the guidance of the kit. The open reading frame sequence of *MfWRKY7* was obtained by reverse transcription polymerase chain reaction (RT-PCR) in combination with specific primers (forward primers: 5′-TCCCCCGGGATGGCGGTTGAGCTTATGTT-3′ (*Sma* I site is underlined) and reverse primers 5′-GGACTAGTCTAAGAAGATTCGAGGACCA-3′ (*Spe* I site is underlined)). PCR product was ligated into the pEasy-Tl Simple vector (TRANSGENE, Beijing, China), and the positive clones identified by PCR were sequenced by Sanger sequencing (Chengdu Qingke Biotechnology Co., Ltd., Chengdu, China). The multiple sequence alignment was performed by DNAMAN9.0, and the phylogenetic analysis was performed by MEGA 11.0 Software. The homologous sequences used were obtained by Blastp search against the NCBI (https://www.ncbi.nlm.nih.gov/ accessed on 1 July 2022) NR protein dataset using the sequence of MfWRKY7 as the query.

### 4.3. Vector Construction and Generation of Transgenic Lines

The amplified fragment of coding sequence of *MfWRKY7* was double-digested by *Sma* l and *Spe* l, and the purified amplicon was inserted into the digested linear DNA of pGSA1403 by T4 DNA Ligase (TaKaRa, Dalian, China). The resulting 35: pGSA1403-MfWRKY7 vector was transformed into *Agrobacterium tumefaciens* strains LBA4404, and the floral-dip transformation method was used to transform the 35: pGSA1403-MfWRKY7 into *Arabidopsis* [47]. T_1_ seeds were selected on 1/2 MS medium with 50 mg/L kanamycin. Homozygous T_3_ lines were obtained by self-pollination of T_1_ and PCR. Two positive lines confirmed by reverse transcription PCR (RT-PCR) were selected for further analysis.

### 4.4. Phenotype Analysis under Drought and Salt Stresses

For seedling treatments, a 1/2 MS medium containing mannitol (0, 200 mM, 250 mM, 300 mM) and NaCl (0, 75 mM, 100 mM, 125 mM) was used, respectively. Sterilized seeds of WT and OE lines were spotted in plates containing the above-mentioned medium with 15 seeds per line per plate. Two days of dark treatment at 4 °C were followed by vertical placement in a light incubator, and root length was photographed and measured after 1 week of normal incubation. Each treatment was repeated three times.

For adult treatments, seeds of WT, OE1 and OE2 were sown in pots with the same weight of the cultivation substrate (soil: vermiculite = 1:1) and incubated in a greenhouse for 4 weeks before drought and salt treatments. For the drought treatment, the WT and OE lines were fully watered first, and then, the watering was stopped. All plants were growing under same condition as described above except that no watering was applied. Plant growth was observed and photographed daily. The salt treatment is similar to that of drought treatment, except that after fully watering, 300 mL NaCl solution (300 mM) was applied in each pot with a 2-day interval. Phenotypic differences between WT and transgenic plants were observed and photographed daily. The experiment was repeated three times.

### 4.5. Measurement of Chlorophyll Content

The 4-week-old plants of WT and OE lines were treated by 200 mM NaCl solution for 2 days and natural drought for 7 days, respectively. Then, 0.5 g of fresh leaves of each line were sampled. The chlorophyll content was extracted using 95% ethanol as described previously [48] and was measured by a spectrophotometer under a wavelength of 649 nm and 655 nm. The 95% ethanol solution was used as a blank control. The experiment was carried out three times with three biological replicates.

### 4.6. Measurement of Water Loss Rate

To measure the water loss rate, rosette leaves of 4-week-old WT and transgenic lines were sampled and weighed (0.5 g). The 0.5 g leaf sample was placed on clean filter paper to dehydrate it naturally under condition of 25 °C and 60% relative humidity. Leaf mass was measured at 0.5 h, 1 h, 2 h, 3 h, 4 h, 5 h, and 6 h, respectively. Three replicates were measured for each treatment.

### 4.7. Physiological Measurements

The proline content was determined by a modified method using the acidic ninhydrin reaction [49]. Malondialdehyde (MDA) content was determined by the thiobarbituric acid method [50]. Soluble protein and soluble sugar contents were determined using the Komas Brilliant Blue method and the plant soluble sugar content assay kit (Nanjing Jiancheng, Nanjing, China), respectively. The accumulation of hydrogen peroxide (H_2_O_2_) and superoxide anion radical (O_2_^−^) in the leaves was illustrated by histochemical staining with 3, 3′-diaminobenzidine (DAB) and nitro blue tetrazolium (NBT) [51], respectively, which was followed by decolorization in 95% ethanol and a photograph. The hydrogen peroxide level and superoxide anion activity were determined by employing the hydrogen peroxide assay kit and superoxide anion kit (Nanjing Jiancheng, Nanjing, China). The superoxide dismutase (SOD) activity and the peroxidase (POD) activity were determined as described previously [52]. Three biological repeats and three technical repeats were executed.

### 4.8. Stomatal Aperture Analysis

Ten rosette leaves of 4-week-old WT and transgenic *Arabidopsis* were sampled and placed in 100 mL of MES-KCl buffer (10 mM MES, 0.1 mM CaCl_2_, 50 mM KCl, pH = 6.15). After 2.5 h of photoinduction, leaves were transferred in 100 mL of MES-KCl buffer containing 300 mM mannitol for 2 h under light treatment. Then, the upper and lower epidermis of the leaves were quickly separated using transparent tape, and the residual chloroplasts on the tape were gently scraped off. The lower epidermis of the leaf was observed and photographed using an optical microscope (DP 80, Olympus, Japan). For the stomatal aperture analysis, about 100 stomata of each sample was measured. Three biological replicates were performed for each strain.

### 4.9. Statistical Analysis

Data from this study were analyzed by Student’s *t*-test in SPSS 23.0. The measured values were expressed as the mean ± standard deviation (SD) of three replicates, and significance of difference was illustrated by * (*p*  <  0.05) or ** (*p * <  0.01).

## Data Availability

Not applicable.

## Supplementary Materials

The following supporting information can be downloaded at: https://www.mdpi.com/article/10.3390/ijms23147890/s1. Ref. [53] is cited in the supplementary materials.
