# Supplementary material for: Heterologous Expression of MfWRKY7 of Resurrection Plant Myrothamnus flabellifolia Enhances Salt and Drought Tolerance in Arabidopsis
Source: Int J Mol Sci. 2022 Jul 17;23(14):7890. doi: 10.3390/ijms23147890 (PMC9324418; doi:10.3390/ijms23147890)
Supplement: Supplementary file 1 [file ijms-23-07890-s001.zip › ijms-1805598-supplementary.pdf]

International Journal of *Molecular Sciences*

MDPI

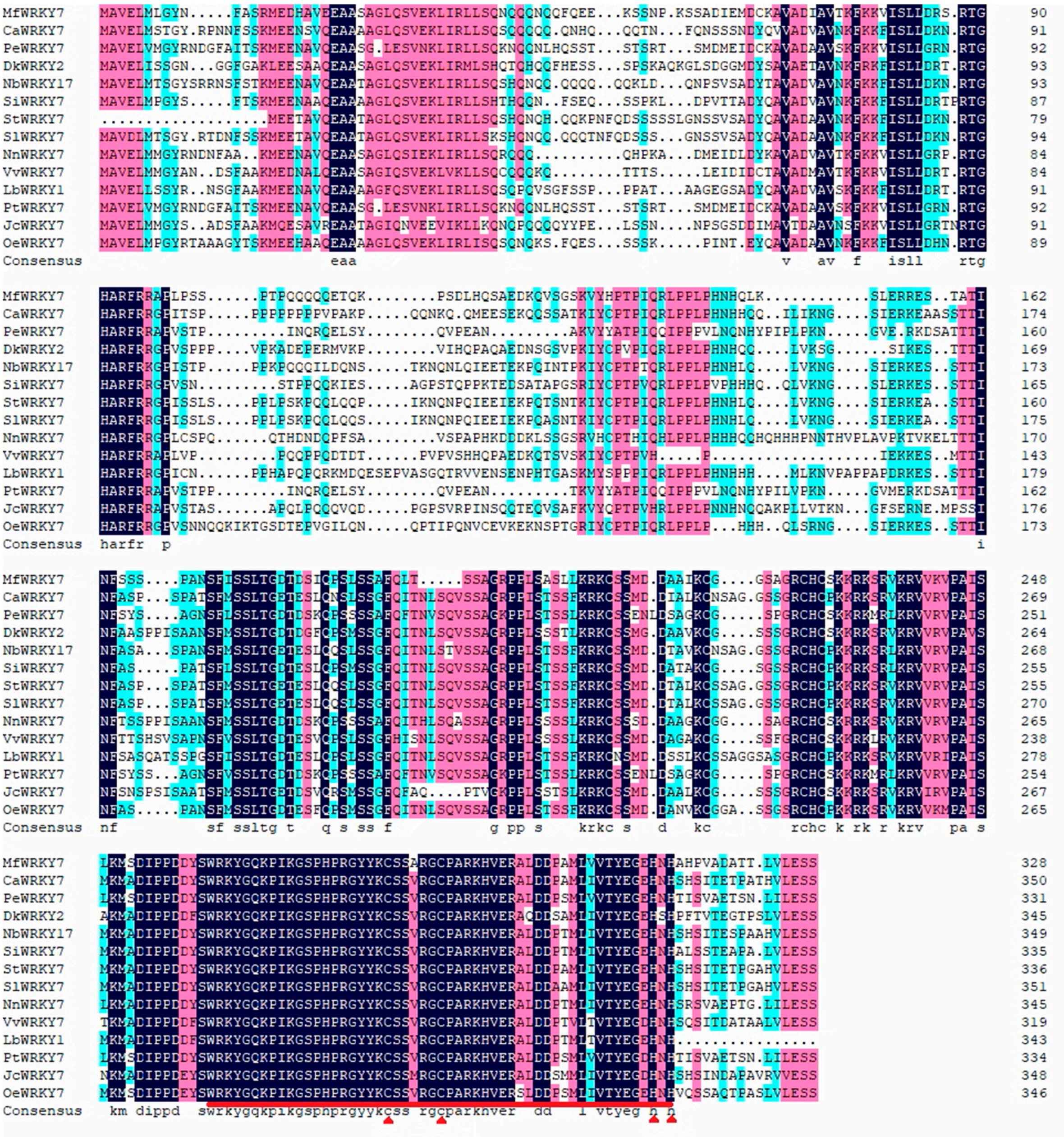

Figure S1 Multiple alignment of deduced amino acid sequences of *MfWRKY7* the some highly homologous WRKYs from different plant species. Identical and similar amino acids are shaded in same color; Red line represented WRKY domain, red triangles represented zinc finger structure, and the altered amino acid residue is indicated by an arrow.

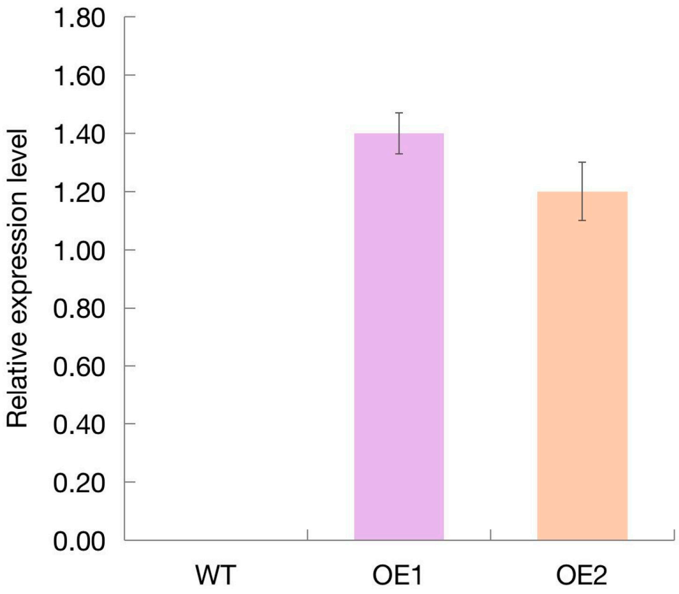

Figure S2 Quantification of expression level of *MfWRKY7* by quantitative real-time PCR (qRT-PCR). Leaves from transgenic and WT plants growing to 4-week-old were sampled. Total RNA was extracted with Plant RNA Kit (Omega Bio-tek, Norcross, GA, United States), which was also purified with RNase-Free DNase I (Omega Bio-tek) and reverse transcribed into cDNA by use of Uscript II (Innovagene biotech, Hunan, China). The qRT-PCR amplification was performed in 25 μl reaction mixture (innovagene biotech) including 12.5 μl 2×Taq SYBR Green qPCR Mix, 0.5 μl of 10 μM of each primer, 4 μl 5-fold-diluted cDNA, and 7.5 μl of Nuclease-free H_2O, which was performed using the real-time PCR instrument CFX Connect (Bio-Rad, Hercules, CA, USA). Amplification conditions were devised that 94°C for 3 min, 42 cycles of degeneration at 94°C for 8 s, annealing/extension at 60°C for 60 s. The relative expression quantity of the target gene was evaluated based on the method of $2^{-\Delta\Delta CT}$[1]. And the results were normalized by an internal reference gene *Actin2* for quantitative analysis of relative genes. Each RT-qPCR experiment was reproduced at least three times. The gene-specific amplification primers were: *Actin2F: 5′*-GGAAGGATCTGTACGGTAAC-3′ and *Actin2R: 5′*-TGTGAACGATTCCTGGACCT-3′ for *Actin2*, and *MfWRKY7F*: 5′-GGGCGCCTTTACCTTCTTCT-3′ and *MfWRKY7R*: 5′-AGCGTTGAATTGGCGTAGGA-3′ for MfWRKY7, respectively.

[1] Livak, K.J.; Schmittgen, T.D.. Analysis of relative gene expression data using real-time quantitative PCR and the 2(-Delta Delta C(T)) Method. *Methods San Diego Calif* **2001**, *25*, 402–408, doi:10.1006/meth.2001.1262.

Table S1 The GenBank accession numbers of the sequences used in Fig. 1b and Fig. S1.

Genes Name	Accession numbers
SlWRKY7	XP_004238130.1_probable
StWRKY2	NP_001275001.1
CaWRKY7	XP_016568783.1
NbWRKY17	AIR74899.1
SiWRKY7	XP_011074403.1
OeWRKY7	XP_022868232.1
LbWRKY1	ACT55331.1
DkWRKY2	AVL95372.1
NnWRKY7	XP_010263444.1
PeWRKY7	XP_011005809.1
PtWRKY7	XP_006380693.1
VvWRKY7	XP_002284966.1
JcWRKY7	XP_020533746.1
